# Medical Society of London

**Published:** 1834-01-01

**Authors:** 


### IV. THE LONDON MEDICAL SOCIETY.

During the present session the London Medical Society has been in great part occupied by discussions on Dr. Tytler's theory, that diseased rice is the cause of cholera. When it is considered that bad rice must have existed in all times, while cholera (in its late form and extent at least,) is a new disease; and that the disease has only within two years been known in many parts of the world, where as much rice was imported formerly as at present; the theory seems so untenable, that one feels some surprise that the society should have tolerated its discussion for four successive evenings. Such, however, was the case, and Dr. Tytler received the thanks of the society, a compliment paid rather to his assiduity and perseverance in supporting his fanciful hypothesis, than to any conviction among the members that he was right.

Oct. 28. Mr. Proctor inquired the opinion of the society about the cholera of this season. He thought it had been modified, and that the treatment generally had been more successful than it was last year, although no specific had been discovered.

Mr. Stephens observed that there were various shades of the disease. He had suffered himself, during the prevalence of the epidemic, from coldness of the extremities, borborygmi, and threatenings of diarrhœa, with numbness, and occasionally even cramps in the limbs. These symptoms were accompanied by a sense of anxiety. Others he had seen in the same state, and he believed that it might be endured some time before the health gave way, and that it did not always lead to cholera, especially if attention were paid to diet.

Dr. Williams gave Mr. Stephens a hint that his own was a case of fright, an opinion which the latter gentleman rebutted; and then arose the interminable dispute whether the recent or Asiatic cholera be a different disease from the ordinary cholera of England, or merely a severer form of it.

Mr. Proctor thought them different diseases, because, in the old cholera, the patients, when they died, sank from the exhausting effects of the vomiting and purging, while the new disease operated at once on the system as a poison. Common bilious cholera, simple diarrhœa, and the Asiatic disease, all existed at the same time as separate diseases.

Dr. Johnson took them to be all grades of the same complaint. The symptoms described by Mr. Stephens were often followed by diarrhœa, and that ultimately by cholera.

Dr. Negri endeavoured to distinguish ordinary diarrhœa from that which precedes cholera. In the latter species there was a strong throbbing pulse, a peculiar expression of the eyes, resembling that of a drunken man in the vascularity of the conjunctiva.

Nothing was said for or against the diagnostic laid down by Dr. Negri, and, in the hands of Dr. Uwins, Dr. Walshman, Mr. Headland, and Mr. Hooper, the question of the identity of the diseases continued to be agitated, without eliciting anything likely to interest your readers.

The meeting, on the 4th of November, was occupied entirely with passing a series of resolutions, commendatory of the conduct of the physicians and surgeons lately attached to the Aldersgate street Dispensary, and which have been elsewhere published.

In the conversation which took place on the 11th, Dr. Uwins, after stating "that the contagions which had been termed specific depended much on time, place, and circumstance, and produced different effects under different states," proceeded to mention some instances tending to prove that the poison of hydrophobia, which was usually traced to one specific source, was subject to modifications; that the rabid poison might be susceptible of graduation, so that an animal in an excited condition, though not positively rabid, might be capable of communicating a disease resembling and even identical with hydrophobia. He next named a case in which insanity had been communicated from a brother to his sister by a bite in the hand.

The last remark excited a good deal of interest in the society.

It was agreed that such an occurrence was extremely uncommon, and Dr. Whiting considered the present instance as an accidental coincidence. The insanity of the sister followed, in his opinion, the insanity of the brother as a natural consequence, if it were a family complaint.

With respect to the spontaneous occurrence of hydrophobia, instances in corroboration were referred to by Dr. Whiting, Dr. Negri, and Mr. Kingdon; but no remarks were elicited which supported in the slightest degree Dr. Uwins' proposition, that hydrophobia is subject to modifications or graduations, (such, however, is probably the fact.)

At the meeting on the 18th, a resolution was passed to the following effect: "That a special meeting of the council be called to *take into consideration the state of the profession*, and to determine on the propriety of calling a meeting of the society to petition Parliament on that subject."

Afterwards, Dr. Uwins drew the attention of the society to some cases of apoplexy, in which bleeding, the usual remedy, was not indicated. The doctor did not appear to us to distinguish these cases by any very well-marked signs from more ordinary instances, in which, however unsuccessful, it is nevertheless the only measure that offers the most distant chance of removing the cerebral congestion. This opinion seemed very general in the society.

Dr. Whiting, Mr. Dendy, and Mr. Headland, all agreed that there were some cases of apoplexy in which mischief might be produced by immoderate bleeding, and they seemed to agree that these cases were usually of the kind denominated serous apoplexy. But little definite was stated as to the symptoms which distinguish the serous from the sanguineous apoplexy, and still less concerning those which indicate the particular serous state in which bleeding proves dangerous.

On the 25th of November, a paper, by Dr. Negri, was read, on the efficacy of secale cornutum in hemorrhage, in leucorrhœa and gonorrhœa. In the introductory part of the paper, the author proceeded to notice the observations of Dr. Atlee, of Philadelphia, Professor Bigieschi, and Dr. Ballardini, in Italy, Dr. Guillemont, in France, and Dr. Marshall Hall, in this country, all of whom had recommended the secale in menorrhagia; of Dr. Shallcross, who employed it in uterine hemorrhage, arising from detachment of the placenta; of Dr. Spajrani, who, in a paper in Omodei's Annals, for March, 1830, speaks of its use in uterine hemorrhage, uterine congestion, epistaxis, hemoptysis, and hematemesis; and of other Italian and French physicians, who have employed it more recently in the same cases. Dr. Negri then observed, that during the time the preceding authors had been investigating the therapeutical powers of the secale, he also had availed himself of numerous opportunities of trying its efficacy in similar cases, and also in gonorrhœa. He had found it of great use, not indeed an infaltible remedy. The dose was five or six grains of the powder, three or four times a day, or oftener.

In the discussion which followed little was elicited, no one appearing to have tried the remedy in similar cases, and all, therefore, being destitute of sufficient experience to speak to any purpose on the subject.

Dr. Shearman raised the objection, that Dr. Negri had not narrated the cases of failure along with those of success.

Mr. Blenkarne thought it had been administered too indiscriminately, and that in the paper just read sufficient distinctions had not been made as to the kind of cases which promised success, and those in which its exhibition might be expected (from what was known of the usual effects of the drug,) to do mischief.

Dr. Whiting considered that to the measures used in some of the cases, in conjunction with the secale, as much might be attributed as to the medicine itself.

Dr. Negri replied that his unsuccessful cases were but three in number, that he had endeavoured to show the particular states which required a different or antiphlogistic treatment; and that, in many instances he had used the medicine alone, in order that its effects might not be confounded with those of other remedies.

At the meeting in December, the following case was related by Mr. Dendy:—A man, æt. 65, who had been subject to scrotal hernia for 25 years, died with symptoms of strangulation. Upon examination, after death, an egg-cup was found in the bowels, with the concave part directed downwards. The discussion turned upon how the egg-cup had been introduced. Mr. Dendy was of opinion (owing to the absence of inflammation in the large intestine, and its presence in the small,) that the egg-cup in question had passed from the mouth, while other members thought that it had been introduced into the rectum.

A special general meeting of the society takes place on Tuesday, the 7th of January, to take into consideration the report of the committee, when the Reform question will, doubtless, be warmly debated.

---

